# A success story of clinical debriefings: lessons learned to promote impact and sustainability

**DOI:** 10.3389/fpubh.2023.1188594

**Published:** 2023-07-05

**Authors:** Méryl Paquay, Robert Simon, Aurore Ancion, Gwennaëlle Graas, Alexandre Ghuysen

**Affiliations:** ^1^Emergency Department, University Hospital of Liege Quartier Hôpital, Liege, Belgium; ^2^Center for Medical Simulation of Liege, Quartier Hôpital, University of Liege, Liege, Belgium; ^3^Retired, Boston, MA, United States

**Keywords:** debriefing, organizational learning, teamwork, quality improvement, clinician wellbeing

## Abstract

The COVID-19 crisis impacted emergency departments (ED) unexpectedly and exposed teams to major issues within a constantly changing environment. We implemented post-shift clinical debriefings (CDs) from the beginning of the crisis to cope with adaptability needs. As the crisis diminished, clinicians voiced a desire to maintain the post-shift CD program, but it had to be reshaped to succeed over the long term. A strategic committee, which included physician and nurse leadership and engaged front-line staff, designed and oversaw the implementation of CD. The CD structure was brief and followed a debriefing with a good judgment format. The aim of our program was to discover and integrate an organizational learning strategy to promote patient safety, clinicians' wellbeing, and engagement with the post-shift CD as the centerpiece. In this article, we describe how post-shift CD process was performed, lessons learned from its integration into our ED strategy to ensure value and sustainability and suggestions for adapting this process at other institutions. This novel application of debriefing was well received by staff and resulted in discovering multiple areas for improvement ranging from staff interpersonal interactions and team building to hospital wider quality improvement initiatives such as patient throughput.

## 1. Introduction

“*Errare humanum est, perseverare diabolicum [‘To err is human, (but) to persist is diabolical']*”. Debriefings emerged from this philosophy of understanding and learning from one's mistakes (1). Developed in the military field during World War II, debriefings have been adopted by several disciplines over the decades (e.g., aviation, psychology, education, and medicine) (2). Debriefings are structured interprofessional meetings, guided by trained facilitators, who aim to promote team reflexivity, learning, and empowerment. These meetings may be characterized by specific semantic elements, such as “after-action review” or “huddle” (3–5). Since their emergence in healthcare, debriefings have mainly been used in simulation-based learning laboratories as initial or continuous training for nurses and physicians. The aim of team training and learning from real critical and complex cases led to a shift from simulation to clinical debriefings (CDs).

Due to the frequent exposure to complex and critical situations, CDs have primarily been introduced and practiced in emergency departments (EDs). Indeed, there is extensive evidence of the benefits of these debriefings in the ED: improvement of knowledge and clinical performance (6–9), communication, team dynamics, and efficiency (9–12), thus impacting patient outcomes (2, 6, 7, 13, 14). These positive impacts, both on patients and the healthcare team, have led to the development of international recommendations advocating for the use of debriefings in the emergency context. As a result, CDs have garnered increased popularity, eliciting enthusiastic support from ED leaders for their implementation (14). However, research in the field indicates that CDs were mainly conducted after critical events and often sporadically or within limited research periods. Additionally, it has been found that these debriefings may have negative effects (3). Therefore, CD implementation remained highly variable for decades (15). The COVID-19 crisis has been a stepping stone to developing new perspectives and potential uses of CD within the ED. The uncertain and constantly changing environment induced by the crisis, considerably challenged, not only EDs but also healthcare institutions, clinical teams, and patient safety. In these difficult and challenging circumstances, CDs emerged as a solution to address many of the patient safety and team adaptation challenges (16, 17).

During this period, studies have investigated the most effective method for conducting CD in the ED and advocated for post-shift debriefings using debriefing with good judgment “plus/delta” method (3, 18–20). The importance of learning not only from failure but also from success, with leadership's wholehearted and visible commitment to act on things that are going well (pluses) and things that need improvement (deltas), appears essential (21). Beyond the question of the art of performing CD, their integration into a global strategy has also been questioned (5). Indeed, by expanding beyond the analysis of specific critical incidents and embracing a broader systemic evaluation of work conducted during the shift, debriefings appeared to hold promise to be a keystone for promoting a learning organization culture and triggering quality and safety improvement (5, 22, 23).

As the worst of the crisis passed, subsequent research seems to have reoriented toward investigating CDs after specific critical events or pedagogical aspects once more. However, questions remain on how to adapt the modalities of these CDs to guarantee their quality and relevance. In that view, moving to “debriefings as a management tool” and making them sustainable required in-depth reflective work. Hence, this article describes the creation of a post-shift-based CD, lessons learned from its implementation, and offers suggestions for adapting this process at other institutions.

## 2. Context: motivations for creating the CD process

The COVID-19 crisis suddenly exposed most EDs to major issues within a fluctuating environment. To cope with adaptability needs, we implemented post-shift clinical debriefings (CDs) at the onset of the crisis. Such CDs proved to be highly efficient and appreciated by the teams. As soon as the clinical situation returned to nearly normal, ED clinicians encouraged ED leadership to rethink ED management in light of lessons learned during the crisis and more particularly the potential use of regular post-shift CDs.

The objective was to develop and integrate an organizational learning strategy within our ED (24):

- to promote quality of care and patient safety
- to promote wellbeing at work by providing space for clinicians to process and reflect
- to empower clinicians and get them engaged.

CDs were implemented in two EDs of a single Belgian University teaching hospital with two geographically separated facilities, namely, Main and Satellite. The Main facility is a tertiary care hospital located in a suburban area, while the Satellite is an urban secondary hospital. The ED from the Main facility was raised under the cultural umbrella of a Public University Teaching Hospital while the second ED history started as part of a private clinic that was merged with the Main Hospital. The two sites combine an annual ED census of ~100,000 patients, with the Main handling ~57% and the Satellite handling 43%. The department employs ~50 physicians and 120 nurses.

## 3. Key programmatic elements: how the CD process was designed and operationalized

During the pandemic, the ED developed a specific process following previously published recommendations for creating a CD program in the ED (13, 20). Upon entering “normal” ED operations, we quickly learned that pandemic-related CDs needed to be revised in a more convenient format and thoroughly integrated into ongoing ED management. Thus, the reshaped program has metamorphized into an effective, well-received management system that is still in use today.

### 3.1. Creating a powerful leadership structure

The chief physician triggered the creation of a specific committee named the Strategic Committee (SC) to support new work strategies. The SC was specifically developed as part of the new initiative and to support new work strategies of the service. The SC was comprised of respected individuals and designated due to their genuine curiosity, influence, and leadership capabilities. In addition, the ED chief physician and the two head nurses were part of the SC. Other ED leaders committed to ensuring follow-up to CD and providing guidance for data management. When the COVID-19 crisis broke out, the chief physician and head nurses naturally joined forces to establish a common strategy and to speak with one voice. At that time, this seemed crucial to avoid conflicting information and decisions. A few physicians emerged as leaders and volunteered to help the SC by monitoring the situation in the field, guiding and implementing decisions, and coaching teams. As soon as the clinical situation returned to nearly normal, ED clinicians encouraged ED leadership to keep the CD program.

Specifically, the SC includes the following:

- The chief physician of the Main and Satellite ED
- The two head nurses from each hospital
- The two assistant head nurses from each hospital
- Five influential physicians, who emerged as powerful resources, were seen as role models by their peers and committed to developing the unit coordination and strategy
- The quality and safety manager.

### 3.2. Identifying a debriefing facilitator, coordinator, and management's role

The quality and safety manager (QSM) position was initiated at the beginning of the pandemic when a call for CD application was launched. The hired QSM, a nurse from another specialty, first spent time becoming familiar with ED processes and teamwork habits and took primary responsibility for the initiative. The QSM had previous experience as a safety manager, was rigorously trained to lead high-quality CDs, and had experience leading simulation debriefings. The QSM works for the ED, with a more transversal role, in connection with the hospital safety department.

### 3.3. Developing the debriefing strategy

Two studies that were carried out during the first wave of COVID-19 laid the groundwork for the process development. The first research described the development and the feasibility of implementing CD during the crisis (20). The second study proposed a framework to categorize the CD content and assess its worthiness (5). Based on these results, it took ~6 months of a quality improvement process to achieve a fully satisfactory integrated CD strategy as detailed below. The objective of these 6 months of continuous improvement was to transition from a crisis context and adapt the debriefing process to a more routine setting. Using Deming's Plan-Do-Check-Act (PDCA) design, different elements were progressively modified to better address the needs of the team and its leaders (e.g., the frequency of debriefings per week, the timing of debriefing sessions, the tools for sharing debriefing outcomes, and the methodology for providing feedback to teams). These modifications were primarily derived from input gathered from teams at the end of debriefing sessions through the QSM, anonymous suggestion boxes for soliciting ideas, and brainstorming sessions conducted within the SC. Moreover, the QSM received guidance and oversight from an internationally recognized expert in ED organization, organizational learning, team management, and change management. It is noteworthy that this principle of continuous improvement is still actively pursued 3 years after the implementation of the process.

#### 3.3.1. Performing the debriefings

Debriefing sessions were performed face-to-face with clinicians (physicians and nurses) twice a week at the end of the shift. Originally, all debriefings were performed by the QSM. During the early months, other nurses and physicians were trained as clinical debriefers. On debriefing days, the debriefer joined the department about an hour in advance to conduct peer check-ins to assess the mood of the team and to promote the post-shift CDs. Those peer check-ins are based on the circle-up framework (16) and include an invitation to talk, use of empathy, exploration, and listening to understand through short prompts (e.g., “How are you feeling right now?” and “How can I support you?”) (16). The debriefer also observed the handover to the next shift to better understand the details of the workflow and clinical status of the unit. Debriefings were held in a private room adjoining the unit to promote access and privacy.

Debriefing began with a quick status check of the team, e.g., “How are you feeling today?” Then, a plus/delta investigation was conducted using short, simple prompts (e.g., “What did you enjoy?” “What challenged you?” “What worked well?” and “What can be improved for next time?”). Participant contributions needed to be as specific as possible. The various pluses/deltas were written down by the debriefer. Then a single delta or plus was chosen to be explored. The CD technique was based on debriefing with good judgment (25). Debriefers captured participants' thoughts on the event using a Frames –> Actions –> Results approach (26). The aim was to better understand the clinical and team thoughts and motivations behind the topic and to explore possible solutions by encouraging team reflexivity. The selected plus or delta was mostly focused on teamwork concepts, e.g., communication, leadership, workload management, and decision-making. Organizational issues or long-term concerns, e.g., institutional bed management, stretcher delays, and faulty equipment, were systematically cataloged (5) and transferred to management for follow-up and typically were not explored during the CD. The exploration of successes turned out to be surprisingly informative and energizing for the teams. The topics that participants appeared most enthusiastic were about personal interactions among team members, starting with concrete examples of interpersonal encounters and then revealing the participant's mental frames and motivations behind their behaviors. Empirically, we noticed that the CD mean duration was ~7 min.

#### 3.3.2. Debriefings analysis and decision-making

Once the CD was over, a brief report was written and included: the date, location, number of participants, CD duration, plus/delta points, and specific suggestions for improvement. Participant anonymity was faithfully maintained in the report. The QSM collected the reports and entered them into the CD database. An updated database categorized all the pluses/deltas raised during CD according to the “Debriefing and Organizational Lessons Learned (DOLL)” (5). The DOLL is a debriefing classification framework that allows the CD to be tracked and systematically integrated with the unit strategy. Issues reported during the debriefings were systematically brought to the attention of the SC, which communicated and executed action plans in coordination with all ED clinicians.

**Figure 1:**
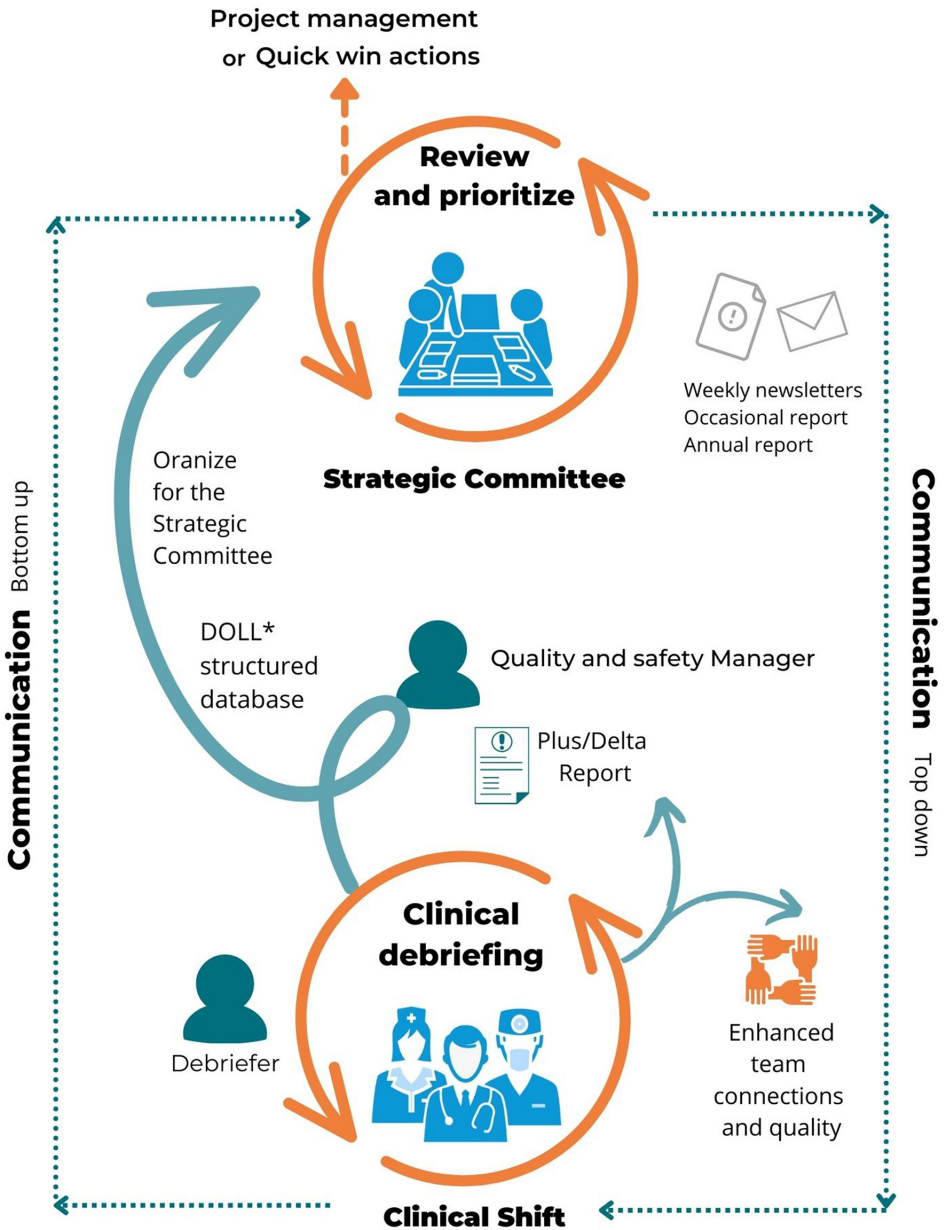
Clinical debriefing as a countercurrent management process. *Paquay et al. (5).

**Figure 2:**
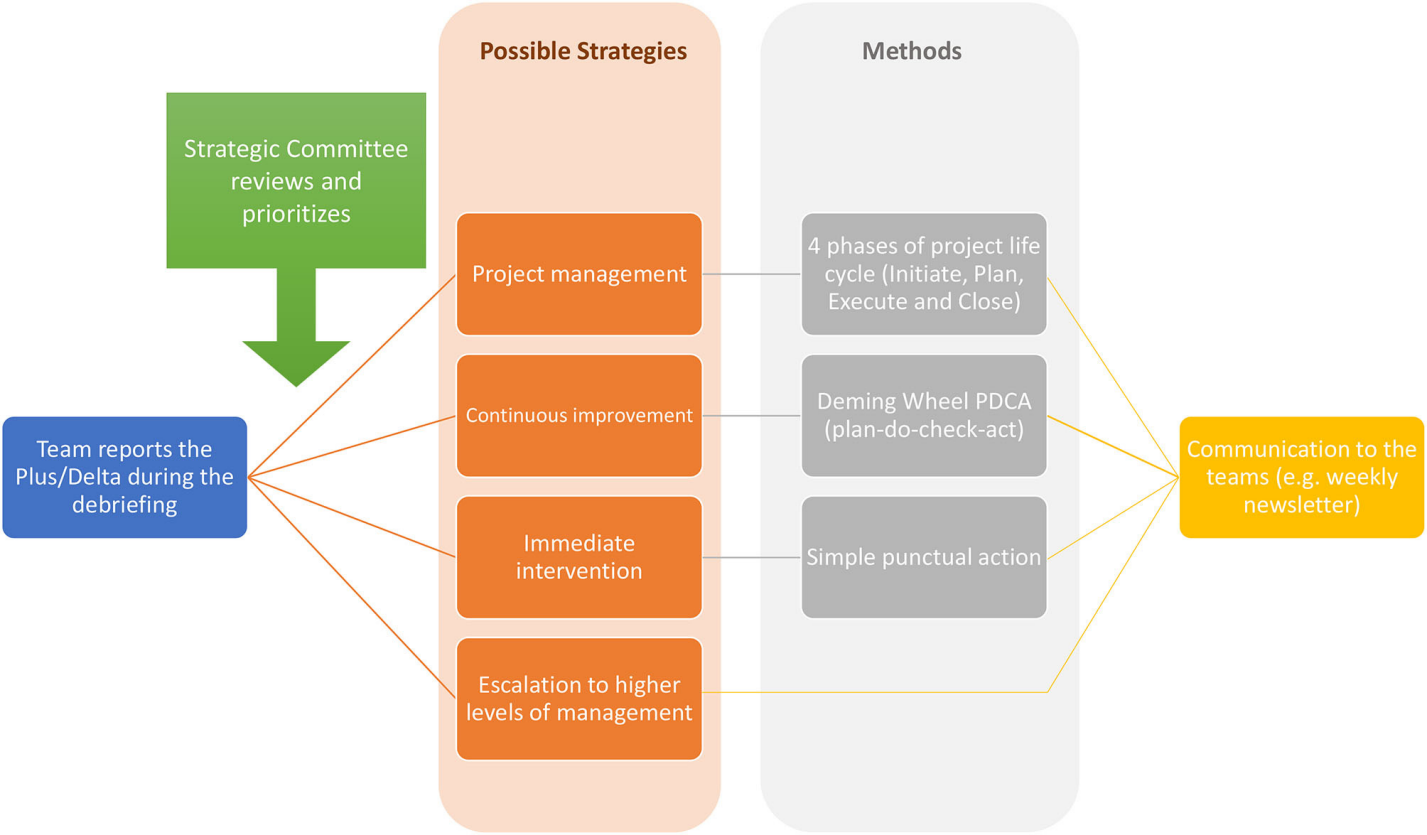
Methodology used based on clinical debriefing subsequent actions.

We identified four ways to deal with debriefing content: “(1) project management using a lifecycle four phases methodology (27); (2) continuous improvement using the Plan-Do-Act-Check process (28); (3) immediate intervention; and (4) escalation to higher levels of management. Table 1 and Figure 1 detail these interventions and action plans (Figure 2; Table 1 give examples of CD content and subsequent actions).

**Table 1:** Examples of clinical debriefing content and subsequent actions.

**Element reported during clinical debriefing**	**Type of subsequent actions**	**What was done**
“There are disparities between nurses and physicians continuing education and nurses feel that is unfair”	Project management	- First was to define and establish of teams' continuing education strategy in the ED - Ongoing
“In general, people are more aggressive in the ED and outside (patients, families, colleagues etc). This is exhausting.”	Continuous improvement	- Multidisciplinary team training focused on conflict management with the collaboration of the psychology department - Charter to promote zero tolerance policy to violence against ED staff - Structural safety initiated, e.g., access control system, camera surveillance, security guards.
“Difficulty in knowing patient's allocation among physicians. Also, some residents have argued the fact they usually get cases nobody wanted”	Continuous improvement	- Started system of random allocation of patients. - The system was assessed and refined through the debriefings following its implementation.
“One nurse was absent, and some physicians didn't know it. Physicians insist on sharing this information during the morning briefing.”	Immediate intervention	- Awareness raised in the weekly newsletter by praising solidarity. - Asking nurses and physicians to systematically ask if some team members are absent.
“Trouble with device batteries. The unit has acquired more and more devices requiring sockets but there are few in the ED.”	Immediate intervention	- Technical department was contacted to add sockets
“Issue related to continuity of care during lunch time. Almost all nurses went together to eat leaving one zone of the unit empty.”	Immediate intervention	- Reminder of lunchtime rules in the weekly newsletter
“Teams are tired of long-term boarding in the ED. Teams explained that the hospital hasn't addressed this problem.”	Escalation to higher levels of management	- The problem has been brought to the attention of the hospital's upper management. - Regular meetings are organized between other departments and ED leadership.

A weekly newsletter was sent by the SC comprised of debriefing points and the status of interventions implemented. While the weekly meetings of the SC were oriented to solving problems and assigning responsibility, emails and small meetings ensued until the issue was ameliorated or solved.

### 3.4. Linking debriefings to existing processes

To move CD from discussion and gripe sessions to a real safety and management tool, creating links with existing quality improvement processes was necessary. The usefulness of the DOLL framework served to illustrate the essence and value of CD. This has been a great support for leaders as it helped them to allocate resources and priorities more efficiently. The grouping of related problems through main categories helped the SC to think in terms of overall quality improvement processes and workflow rather than focusing on the previous method of singular problem solution. The ED found itself collectively developing ongoing quality improvement programs *vis-à-vis* the CD coupled with favorable action. When using the framework, more links were made between CD and their evolving content over time.

## 4. Practical implications and lessons learned for future applications

### 4.1. Numeric results

It should be noted that we have not attempted to directly link CD to quality and organizational metrics. To provide a numeric overview, below are numbers to help the reader understand the size of the program.

From April 2020 to December 2021:

- 273 debriefings were performed
- 978 items were identified
  ° 355 pluses
  ° 623 deltas

- 66 strategic meetings of the SC were performed

Delta management:

- 60% were solved by a simple action/change within the week of the debriefing.
- 15% required a long-term project with a specific action plan.
- 15% have been solved through ED team information and awareness.
- 10% are outside ED scope and have been assigned to various hospital processes for improvement.

Patient safety indicator:

- Compared to previous years, incident reports were increased by 53%.
- Also, we observed an increase (3%) in the incident reports among the medical team. Before that, incident reports were mostly done by the nursing team.
- The perceived quality and safety culture has increased. We believe that this improvement was not only the result of the CD but was also part of a nascent positive culture change. Indeed, we sense and feel that the enhanced culture was built upon two key elements happening during CD, namely, genuine curiosity and shared reflective practice. These, in turn, authorized speaking up, reporting, and reflective thinking (24). We feel that this brought trust, confidence, and an *esprit de corps* to the ED.

### 4.2. Lessons learned

- At the start of the project, we took the attitude that “best is the enemy of good.” We wasted some time trying for the “best” and then decided to start small and apply quality improvements along the way. This approach was communicated to the department and asked for their patience and support as the program matured. Over time, synergies and improvements emerged naturally. QI implementation should be seen as an ongoing process operating on the basis of clinician feedback loops.
- When CDs were launched, some clinicians thought that the initiative was part of the hospital's accreditation process. Teams reported being less motivated to conduct debriefings in that context. Once the teams understood that the initiative was being implemented by and for them and had the full endorsement of the ED leadership, legitimacy and motivation were enhanced.
- At first, we thought that debriefers should already be simulation instructors. A few clinicians had a 1-year certification in medical simulation. Those were the first training with a 1-day clinical debriefing training and *in situ* coaching by the main debriefer (QSM). With time, we have observed that practice in medical simulation was not necessary. Values of simulation (e.g., good judgment, using the basic assumption, ensuring psychological safety, respectfully handling difficult conversations, and effectively managing emotions were core competencies for clinical debriefings and could well be learned in the CD context.
- Team members were less motivated by scientific findings about CD but were motivated by their personal observations of changes and improvements in the workplace. However, ED leadership required scientific evidence and impact on patient safety.
- Teams' desire to change can fluctuate. During the COVID-19 crisis, the context was extremely favorable to rapid, ED-wide change implementation (more “immediate interventions” rather than “project management”). Issues were concrete, and the solutions were provided within days. Once the intensity of the crisis subsided, the change process slowed and there were several complex issues raised for which leadership did not have near-term solutions. This was demotivating. These larger problems require patience, strategic thinking, and often interdepartmental cooperation. With more experience, we noticed that exploring these points during the CD led to frustrating complaint sessions.
- The solution was to record every problem during the plus/delta portion of the debriefing. The debriefer then focused on topics the team could control, e.g., conversations and team coordination, mutual support, and communication issue. After the CD, all issues that required more thought, multi-department coordination, and planning to implement, e.g., failure to rapidly make empty in-hospital beds available, were dutifully reported to the SC for their consideration and cross-department action.
- A major obstacle for nurses was to have the CD after their shift hours. A strategy was then organized so CDs were held before the end of the shift. Moreover, debriefers committed to respect the end-of-shift hour. If the discussion took a longer time, it was stated that the CD was going to exceed the end-of-shift hour and participants were offered a choice to continue or close. This approach improved participation.
- In the beginning, the debriefer appeared in the ED just before the scheduled CD. We learned that some valuable information was learned by a short observation before CD. To make better use of valuable clinician time, the lead debriefer went to the ED with plenty of time to “take the pulse” of the ED, i.e., the mood, significant events that may have occurred, how someone seemed out of sorts, etc. These early appearances also allowed the debriefer to have follow-up discussions with the clinicians about past concerns. This approach solved several problems, i.e., saved time, the debriefer was considerably better informed and often had an idea of the most worthwhile topics, and demonstrated the debriefer's personal involvement and commitment.
- Leaders were discouraged at the beginning because many issues required complex problem-solving and institutional coordination. To address this issue, we decided to prioritize actions. Issues related to institutional long-term situations were reported and tracked as appropriate but not given a high CD priority because they required quite a bit of time to address. On the other hand, teamwork, communication, and enhancing mutual respect were interesting and motivating topics for participants.
- At the outset, CD tended to focus on deltas, which were unpleasant and dispiriting by their very nature. Debriefers altered their approach and became skillful also at having interesting learning conversations using plus actions by clinicians.
- Healthcare quality initiatives have a reputation for starting and then slowly dying. Experienced staff resist initiatives partly for that reason, E.G., “If I wait long enough this program will go away like the rest of them do.”: “this program won't make any difference in my clinical lift [SIC].” This lack of clinician enthusiasm was entrenched and discouraging. To overcome this common problem, (a) ED leadership visibly committed to the program being a long-term/permanent quality assurance technique. (b) Made clear to clinicians that we knew that the program was not perfect and that ED leadership was committed to refining and improving the innovation as experience increased. (c) Committed to public updates on project problems and project improvements and to providing public examples and project successes. In short, we publicly committed to “never give up.”

### 4.3. Suggestions for starting a clinical debriefing program

The first steps to get started with CD are as follows:

- Have visible management support and engagement: In our case, the development of the SC, with the chief physician and the head nurses speaking with one voice regarding CD and using CD as part of their management strategy.
- Provide a sustainable resource to coordinate all aspects of the process: one specific person (e.g., in our case, the QSM) should be responsible for the quality and sustainability of the CD process. Clinical debriefing coordination should be included in a function definition.
- Regarding roles and competencies for selection, influential physicians should be individuals esteemed as paragons by their peers, exhibiting a heightened comprehension of unit functioning, while possessing adeptness in team management and task delegation. The CD coordinator should have profound and intricate comprehension of team management principles, change management strategies, and process management methodologies. Debriefers should cultivate the values of simulation (curiosity and sound judgment), emotional management, and handling difficult conversations. These debriefers must primarily undergo training and be coached by an experienced debriefer in these concepts.
- Develop an internal process for structuring and managing data, e.g., the DOLL or something like it.
- Establish department-wide regular communication regarding the program, e.g., a weekly newsletter, specific emails, department meetings, and annual reports.
- Celebrate success and give credit for good ideas.
- CD coordinators (e.g., QSM) and initiators (e.g., SC) should stay consistent, keep a positive vision of debriefings (e.g., publicly support the process, highlight when a change has been made thanks to CD, and provide regular feedback), and never give up on a commitment to making this work. Do not let the naysayers “win.”
- The initiative should come from the unit leaders (e.g., head physician and head nurse) and be clearly explained to the teams.
- CDs should be integrated into the unit strategy and associated with other existing processes (incident reports, complaints, etc.). This is our next step.
- The CD process should be adapted to the needs, time, and experiences of the unit, i.e., the number of times CDs take place might fluctuate from every day to twice a week. This may fluctuate, but do not quit!
- Get started and make it better with time. Let the department know that is the approach. Adapt to the unique circumstances in a department.
- Listen to the outspoken critics. Be curious and respectful of criticisms.
- Unit leadership and the program leader should be aware of the scientific evidence on CD. Share it when clinicians show an interest.
- Debriefers should be familiar with unit daily operations and organization.
- Communication flows should be established. Consider who needs what information: clinicians, action committee, unit leadership, and hospital leadership.
- Teams must feel and see visible changes in their everyday work (dashboard, newsletter, follow-ups, etc.).

### 4.4. Suggestions for having interesting conversations

CD should:

- Start with quick *highly specific* examples using a plus/delta method.
- As a minimum, include nurses and physicians.
- Be brief with a maximum of 15 min for the CD.
- The CD should be proximal to the unit and preferably not in an active clinical space.
- Include a trained debriefer who is experienced in handling difficult conversations with respect and a willingness to share points of view.
- Focus on interpersonal, teamwork, and organizational issues rather than equipment, strategic, and hospital issues.
- When going deeper into plus or delta, think about asking follow-up questions because the first answer is likely to be superficial. Probe deeper.
- Debriefers can respectfully insert their own opinions for examination by others.
- CD reports should synthesize the plus/delta and be shared with leaders.

## 5. Methodological constraints

Only descriptive statistics were performed to summarize the frequencies and percentages of the pluses and deltas in each dimension of the DOLL as well as subsequent actions.

As part of the CD continuous improvement process, qualitative data were collected through diverse formal and informal means:

- Clinicians and SC members have been surveyed through individual interviews and focus groups.
- Debriefing content was also analyzed to evaluate the whole process.

As the DOLL classification framework and its implementation were based on these data, further research is needed to test the model in different localities and contexts. Interpretation bias is also common in qualitative studies during data collection and analysis. Currently, the successful integration of CD into quality and safety processes, unit coordination, and human resources is under consideration. Indeed, the extent of the impact resulting from this integration on patient safety variables and staff wellbeing remains to be fully performed and comprehensively assessed over a long-term period.

## Data availability statement

The original contributions presented in the study are included in the article/supplementary material, further inquiries can be directed to the corresponding author.

## Ethics statement

Ethical review and approval was not required for the study on human participants in accordance with the local legislation and institutional requirements. Written informed consent from the participants was not required to participate in this study in accordance with the national legislation and the institutional requirements.

## Author contributions

MP conceived and designed the study, realized data analysis, and wrote the first draft. GG and RS helped with study design, facilitated data transcription, and performed data analysis and interpretation. AA performed data analysis and made critical revisions. AG conceived and designed the study and performed critical editing supervision. All authors contributed to the manuscript revision, read, and approved the submitted version.
